# Probing Evolutionary Repeatability: Neutral and Double Changes and the Predictability of Evolutionary Adaptation

**DOI:** 10.1371/journal.pone.0004500

**Published:** 2009-02-23

**Authors:** Scott William Roy

**Affiliations:** National Center for Biotechnology Information, National Library of Medicine, National Institutes of Health, Bethesda, Maryland, United States of America; The Research Institute for Children at Children's Hospital New Orleans, United States of America

## Abstract

**Background:**

The question of how organisms adapt is among the most fundamental in evolutionary biology. Two recent studies investigated the evolution of *Escherichia coli* in response to challenge with the antibiotic cefotaxime. Studying five mutations in the β-lactamase gene that together confer significant antibiotic resistance, the authors showed a complex fitness landscape that greatly constrained the identity and order of intermediates leading from the initial wildtype genotype to the final resistant genotype. Out of 18 billion possible orders of single mutations leading from non-resistant to fully-resistant form, they found that only 27 (1.5×10^−7^%) pathways were characterized by consistently increasing resistance, thus only a tiny fraction of possible paths are accessible by positive selection. I further explore these data in several ways.

**Principal Findings:**

Allowing neutral changes (those that do not affect resistance) increases the number of accessible pathways considerably, from 27 to 629. Allowing multiple simultaneous mutations also greatly increases the number of accessible pathways. Allowing a single case of double mutation to occur along a pathway increases the number of pathways from 27 to 259, and allowing arbitrarily many pairs of simultaneous changes increases the number of possible pathways by more than 100 fold, to 4800. I introduce the metric ‘repeatability,’ the probability that two random trials will proceed via the exact same pathway. In general, I find that while the total number of accessible pathways is dramatically affected by allowing neutral or double mutations, the overall evolutionary repeatability is generally much less affected.

**Conclusions:**

These results probe the conceivable pathways available to evolution. Even when many of the assumptions of the analysis of Weinreich et al. (2006) are relaxed, I find that evolution to more highly cefotaxime resistant β-lactamase proteins is still highly repeatable.

## Introduction

How predictable is evolution? Adaptive evolution is unlikely to be strictly deterministic, owing to a number of factors including the stochasticity of evolution in finite populations and epistasis. On the other hand, the finite universe of genetic possibility, shared ancestry, and interactions among traits and loci also constrains the number of possible evolutionary trajectories and outcomes, and widespread convergent evolution of morphological and genetic traits may suggest the existence of a small number of available solutions to some evolutionary problems.

An elegant test of evolutionary repeatability was recently reported by Weinreich et al. [1] The authors studied a set of five mutations in the *E. coli* β-lactamase gene whose co-occurrence confers strong resistance to the antibiotic cefotaxime [2]. Weinreich et al. [1] constructed all 2^5^ = 32 distinct haplotypes (this is, the wildtype, all five single mutants, all ten double mutants, etc.), and tested each haplotype's level of resistance to antibiotic. If all five mutations increased resistance on all genetic backgrounds, there would be 120 (5×4×3×2×1) possible five-step pathways leading from wildtype to the fully resistant haplotype, consisting of the five single mutational changes in all possible orders. Instead, they found that the effect of some mutations on antibiotic resistance was strongly dependent on genetic background – of the five mutations, only one increased resistance on all 16 possible genetic backgrounds, and three of the five actually decreased measured resistance on some backgrounds. In all they found that for only a relatively small fraction (15%, 18/120) of possible pathways, all five steps increased resistance, suggesting that a relatively small number of possible pathways are accessible to natural selection. The authors then extended the analysis to include the possibility of mutational reversals during adaptation, which increased the number of accessible pathways by 50% [3]. Throughout, ‘possible’ pathways refers to all formally possible pathways from wildtype to fully resistant haplotype under the allowed types of mutational changes (i.e. forward, back, double). ‘Accessible’ pathways refers to the subset of possible pathways that are accessible under the specified selective rules (i.e. beneficial, neutral).

Here I extend this analysis in various ways. Increasing the palette of allowed changes between haplotypes – by allowing for multiple simultaneous changes – greatly increases the number of accessible pathways. Increasing the range of permitted phenotypic changes – by also allowing ‘neutral’ pathway steps that do not change antibiotic resistance – also considerably increases the number of accessible pathways. However, the true effect of such changes on the overall predictability of evolution is less clear. I introduce the concept of pathway ‘repeatability,’ allowing for direct comparison of evolutionary predictability over different sets of assumptions. Pathway repeatability is generally less affected by allowing for additional classes of changes than is total number of accessible pathways.

## Methods

I obtained the data from the initial publication of Weinreich et al. from the authors. The resistance of one haplotype (A42G+M182T) was altered in line with the findings of a subsequent study (MIC = 0.5 µg/ml, Kyle Brown, unpublished results). Following Weinreich and DePristo and coauthors [1], [3], two selective models were used. Under truncating selection, all haplotypes with resistance greater than the current haplotype are assumed to have equal selective advantage, thus any de novo resistance-increasing mutation has equal probability of fixation. Under the ‘EVT’ model [4], [5], changes that cause larger increases in fitness are assumed to have greater fitness, thus different de novo resistance-increasing mutations have different probabilities of fixation. See DePristo et al. [3] for details. Overall, the probability of a pathway consisting of *TEM* ^1^ →A→B→C→D→TEM* is given by the product of the probabilities the individual steps: *P*(*A→B*)×*P*(*B→C*)… etc. All analyses were performed by novel PERL scripts.

## Results and Discussion

### Repeatability

Following Weinreich and DePristo and colleagues [1], [3], I characterize the distribution of accessible pathways in two related but separate ways: by looking at the total number of accessible pathways, and by looking at the distribution of probabilities among these pathways. These two ways of looking at the question measure very different things: for instance, even a large increase in the number of accessible pathways may not greatly alter the distribution of probabilities among accessible pathways, if many new pathways have very small probabilities. Here a useful metric is: the probability that two random trials follow the same pathway, which I call ‘path repeatability.’ As the probability that any given pathway is observed for both of two random trials is just the square of that pathway's probability, total repeatability is equal to the sum of the squares of the probability of each pathway, summed over all accessible pathways.

Weinreich et al. [1] considered two selection models, with potential next steps in a pathway having either equal probabilities of occurrence (“truncating selection model”) or with more resistant haplotypes having greater probabilities, with different probabilities given by Extreme Value Theory (“EVT model”). Under the truncating selection model, among the 18 accessible forward five-step pathways, two pathways account for 29.2% of the total probability (that is, a random trial has a 29.2% chance of having one of those two path outcomes). Overall, the path repeatability is 7.8%. While allowing single back mutations increases the number of accessible pathways by 50% (to 27), path repeatability is only mild affected, decreasing to 7.1%. Under the EVT model, the effect is even smaller, with path repeatability equal to 20.94% without back mutations and 20.90% allowing back mutations.

### Types of mutations

In their initial analysis, Weinreich et al. [1] considered forward, single, beneficial mutations. The first restriction was then relaxed in their follow-up paper, which allowed for mutational reversals in the path (that is, multiple changes at the same site, hereafter ‘back mutations’ [3]). Here, I consider the effect of allowing additional types of genetic change, by allowing adjacent haplotypes along a pathway to differ at two sites (double mutations), and by allowing neutral changes (those that do not change the measured level of resistance) along a pathway.

### Neutral mutations

I first studied the effect of allowing neutral as well as beneficial mutations. The importance of neutrally selected changes in the evolution of gene sequences has long been appreciated, and the possibility of long series of changes along potentially vast neutral networks of interconnected genotypes has been extensively discussed before [6], [7].

Allowing only single forward mutations, there are 5! = 120 possible pathways, of which 18 were found to be accessible by beneficial mutations alone [1]. Allowing neutral mutations increases the number of accessible pathways by four fold, with 78/120 possible pathways now accessible (Table 1; note that throughout this section on neutral mutations, pathways are only allowed to visit the same haplotype once, since otherwise an infinite number of possible pathways could be generated by different numbers of alternations between haplotypes of equal fitness).

**Table 1:** Numbers of pathways and path repeatability under different conditions.

Backwards	Double (Relative LH)	Neutral (Relative LH)	Model	Number of Pathways	Repeatability (%)
No	No	No	Truncating	18	7.8
Yes	No	No	Truncating	27	7.1
No	No	No	EVT	18	20.9
Yes	No	No	EVT	27	20.9
No	No	Yes	Truncating	78	
		1% (Nes = 50)			7.4
		5% (Nes = 10)			6.2
		100% (Nes<1)			1.9
Yes	No	Yes	Truncating	629	
		1% (Nes = 50)			6.7
		5% (Nes = 10)			5.5
		100% (Nes<1)			1.4
No	Yes	No	Truncating	147	
	1%				6.6
	5%				3.6
	100%				1.3
No	Yes	No	EVT	147	
	1%				15.0
	5%				5.3
	100%				3.7
Yes	Yes	No	Truncating	4800	
	1%				5.8
	5%				2.9
	100%				0.4
Yes	Yes	No	EVT	4800	
	1%				15.0
	5%				5.3
	100%				2.9

The effect of neutral mutations on the number of accessible pathways is even more dramatic when back mutations are allowed. DePristo et al. [3] enumerated more than 18 billion possible pathways including at least one back mutation, of which only 9 were characterized by steadily increasing resistance (i.e. 27 total accessible pathways allowing only beneficial single changes). Allowing neutral changes increases this number by 60 fold, to 551 (629 total pathways; Table 1). Thus allowing for neutral changes both increases the number of total accessible pathways and the proportion of accessible pathways that include a back mutation (from 33.3% to 87.6%).

How does allowing neutral mutations affect pathway repeatability? The answer depends on the probability of fixation of neutral mutations relative to beneficial ones. In general, the probability of fixation for a newly arising mutation is roughly 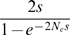 in a haploid population [8] (which is roughly 2*s* for a beneficial mutation), and 1/*N_e_* for a neutral mutation. Thus the relative probability of fixation of a neutral mutation relative to a beneficial one is 1/2*N_e_s*.

**Figure 1:**
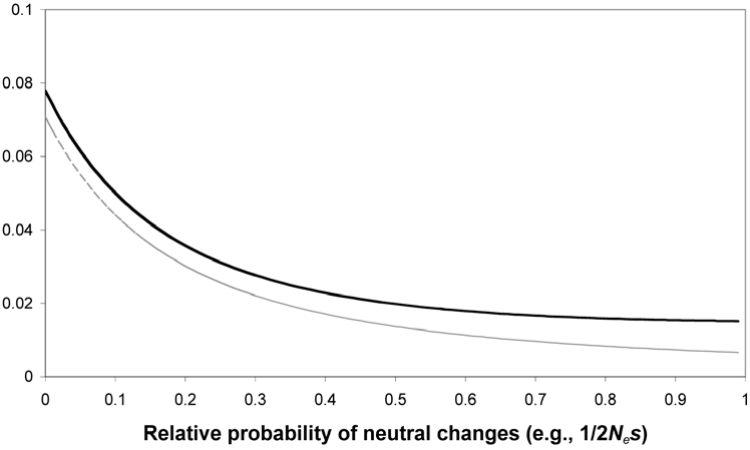
The influence of neutral changes on path repeatability. Path repeatability is shown as a function of the probability of neutral changes relative to beneficial mutations (which are assumed to have equal probability), which is roughly equal to 1/2*N_e_s* in a haploid population. Results are shown both excluding (upper trace) and including (lower trace) back mutations.

So long as neutral changes are much less likely than beneficial ones, repeatability is not greatly affected – under truncating selection, assuming that neutral mutations are 1% as likely as favored mutations (for instance when *N_e_s* = 50) reduces repeatability by around 5% either allowing (7.8% to 7.4%) or not allowing (7.1% to 6.7%) back mutations. If neutral mutations have 5% the probability of favored ones (*N_e_s* = 10), repeatability reduces by around 20% (to 6.2% and 5.5% without/with back mutations). On the other hand, when neutral mutations are roughly equally as likely as favored ones (i.e., *N_e_s* less than 1), repeatability decreases much more, by around fourfold for equal probabilities (to 1.9% and 1.4%). The effect of relative neutral mutation probability on repeatability is illustrated in Figure 1.

### Double changes

Next, I studied the effect of allowing double changes. Again our starting point is allowing only single beneficial forward mutations, in which case 18/120 pathways are accessible. I then allow double changes – that is, I allow for direct transitions between haplotypes that are separated by differences at two sites. Allowing double changes (but not yet back mutations), there are 240 total possible paths with one double mutation, and 90 additional possible paths with two double mutations. Of these, I found 31.7% (76/240) of possible paths with one double mutation and 58.9% (53/90) of possible paths with two double mutations were accessible allowing only beneficial changes. Thus allowing double mutations significantly increases the total number (147 versus 18) and fraction (147/450 = 32.7% versus 18/120 = 15.0%) of possible paths that are accessible by beneficial mutations.

**Figure 2:**
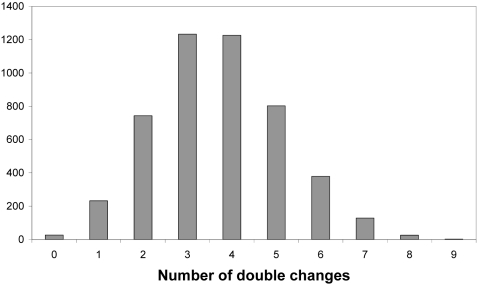
Number of double changes and the number of pathways. For each number of double changes, the number of accessible pathways with that number of double changes (allowing back mutations) is given.

As with allowing neutral mutations, the effect of allowing double mutations on the number of accessible pathways is more pronounced when back mutations are allowed. As compared to the 27 accessible pathways allowing only single beneficial forward and back mutations, there are 232 accessible pathways allowing one double mutation along a given pathway, and 4800 accessible pathways allowing multiple double mutation events in a pathway. Interestingly, the number of total accessible pathways shows a sharply peaked distribution relative to the number of total double changes, with 83.4% of possible accessible pathways having between 2 and 5 double changes (Figure 2).

**Figure 3:**
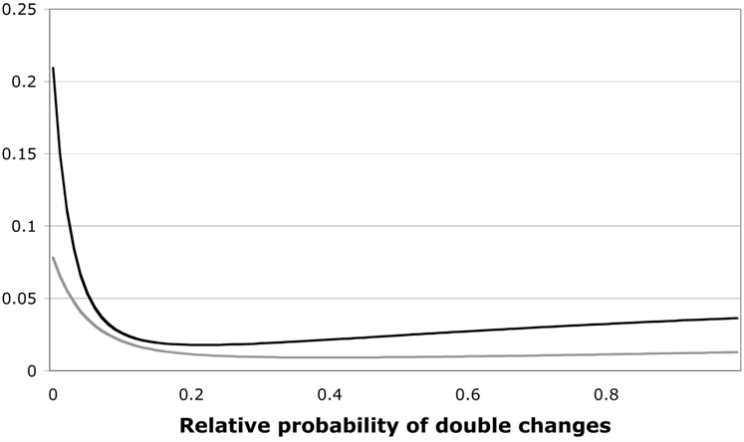
Probability of double changes and pathway repeatability. Path repeatability is shown as a function of the probability of double changes relative to single changes for forward mutations under both EVT (top trace) and truncating selection (bottom trace).

Again, the effect of these additional types of changes on repeatability depends on their relative probability. As with neutral mutations, if double mutations have a small probability relative to single mutations, repeatability is not drastically affected. Under either truncating or EVT selection, repeatability is reduced by around 15–25% when double mutations are 1% as likely as single mutations, and by around 60–75% when double mutations are 5% as likely. Interestingly, in contrast to the case with neutral mutations, repeatability does not strictly decrease with the relative probability of double mutations, but begins to increase when double changes are at similar probabilities to single changes (Figure 3). The explanation for this observation appears to be that as double mutations become likely, a few very short paths including one or two double changes reach high probabilities, contributing a large amount to repeatability.

### How important are neutral and double mutations?

In their previous analyses of the empirically derived fitness landscape studied here, Weinreich, DePristo, and coauthors restricted themselves to single beneficial changes [1], [3]. This was due to the so-called ‘strong selection, weak mutation’ assumption, that is, that adaptation occurs through emergence and rapid fixation of single mutations. This assumption disregards the possibility of double mutants, including the possibility that even negatively selected individual mutations will be segregating at low frequencies in the population allowing for the emergence of such double mutants.

One of the most compelling theoretical arguments for the importance of double mutations in adaptation comes from Weinreich and Chao [9], who showed that in large populations double mutations will be sufficiently likely to allow escape from local maxima. However, that case is an importantly different case than the present one. As local maxima are (locally) stable, a population may spend a large amount of time at a local maximum, allowing for the accumulation of both neutral and slightly deleterious variation within the population, as well as fixation of (nearly) neutral mutations by drift. Indeed, as multiple mutations are required to escape from local maxima, in the theoretical case even the rarest combinations of mutations will have sufficient time to accumulate in the absence of competing mutational combinations.

The case here is quite different. At the moment that selective pressure is first applied, there may be standing variation allowing for the possibility that the first successful beneficial variant could be a double mutant relative to the ancestral type. However, in the absence of significant recombination, this initial sweep will wipe out this standing variation. Thus if selection is sufficiently strong, subsequent sweeps are likely to begin before significant variation has accumulated within the population, greatly reducing the possibility that the second sweep will involve a double mutation.

However if selective pressure is not constant, the potential for variation and or drift leading to subsequent sweeping haplotypes to have multiple differences from each other is increased. In one simple case, if selection is intermittent, a haplotype that is driven to fixation during one episode of strong selection may experience subsequent changes during a period of relaxed selection (these mutations may increase, decrease, or not affect resistance), in which case the eventual path taken may include non-favored and/or double mutations. Another possibility involves strong truncating selection with gradually increasing selective pressure – in this case all haplotypes that can withstand a given concentration will have equal fitness at that concentration regardless of their ability to withstand higher concentrations. Then, a haplotype that sweeps at a given concentration may subsequently undergo additional resistance-neutral or resistance-reducing mutations. If antibiotic concentration then increases, the eventual victor in the second step may thus include intermediate mutations that did not increase resistance.

For instance, consider a period of neutral evolution beginning with a genotypically homogenous population (e.g., following a sweep), modeling mutation as a Poisson process. After a time *t* (small relative to the inverse of the mutation rate), the probability of an individual having a particular haplotype that is one step away from the previous one (e.g., the sweeping haplotype) will be roughly the probability that a given site has changed (1-*e* ^−*tu*^) times the probability that the other four sites have not, *e* ^−4*tu*^, where *u* is the rate of mutation per unit time per site. The probability of an individual having a haplotype that is two steps away will be roughly (1-*e* ^−*tu*^)^2^ times *e* ^−3*tu*^. If sufficient variation accumulates during this neutral period, followed by another period of selection, the ultimately successful haplotype will likely be drawn from the standing variation. From the above, after a time *t* of neutral evolution the frequency of a double mutant relative to a single one is (1-*e* ^−*tu*^)^2^ *e* ^−3*tu*^/[(1-*e* ^−*tu*^)*e* ^−4*tu*^] = *e^tu^*-1, which is roughly *tu* for small *tu*. Thus the relative probability of double mutants relative to single ones (as in Figure 3) may be interpreted as roughly the amount of mutation per site during a period between selective episodes. The probability of neutral mutation may similarly be interpreted as the amount of mutation between sweeps, though instead of selective pressure being relaxed entirely during the interim period, in this case selection would be sufficient to prevent decreases in resistance, but not to favor further increases.

### The effect of the fitness of individual haplotypes

I also studied the effect of individual haplotype fitnesses on pathway accessibility. In the initial dataset, some haplotypes were appropriately scored as having equal resistance, as the assay did not allow for distinguishing between haplotypes with similar resistance profiles. However, these haplotypes are unlikely to have truly identical resistance. If so, some favored steps (even if only mildly so) have presumably been treated as neutral. Weinreich et al. found only a modest effect on overall path number of randomly breaking these ties only including forward mutations (see the supplemental materials of ref. 1). Here, I consider the case when back mutations are allowed.

In the current data set, there are five ties ranging from a two-way tie to a seven-way tie. Overall, the five are 2,3,4,6, and 7-way ties, thus there are 7!6!4!3!2! = 1.05 billion ways to break the ties. To explore the effect of these ties on the number of accessible paths, I performed 10,000 simulations, in each case randomly breaking each tie. The number of accessible paths for these tie-breaking trials varied widely. In some cases, breaking ties only slightly increased path number, to 30 total pathways (compared with 27), whereas in others the number of accessible pathways was increased to up to 298 total pathways. Overall, the mean/median number of pathways was 76.8/74 across trials. Thus breaking ties in this way increases the number of accessible pathways including back mutations more than previously found by Weinreich et al. excluding back mutations.

### Concluding remarks

Weinreich et al. broke considerable ground in their systematic treatment of the context-specific effects of different mutations in protein evolution. The current results extend the previous studies of Weinreich and DePristo and colleagues. With the increasing availability of high-throughput technologies, mapping of additional fitness landscapes should allow for the testing of the generality of the previous and present conclusions.
